# Estimation of Long‐Term Efficacy of Denosumab Treatment in Postmenopausal Women With Osteoporosis: A FRAX‐ and Virtual Twin‐Based Post Hoc Analysis From the FREEDOM and FREEDOM Extension Trials

**DOI:** 10.1002/jbm4.10348

**Published:** 2020-02-24

**Authors:** Ethel Siris, Michele McDermott, Nicola Pannacciulli, Paul D Miller, E Michael Lewiecki, Roland Chapurlat, Esteban Jódar‐Gimeno, Shuang Huang, John A Kanis

**Affiliations:** ^1^ Dept of Medicine Endocrinology, Columbia University Medical Center New York NY USA; ^2^ Amgen Inc. Thousand Oaks CA USA; ^3^ Colorado Center for Bone Research Lakewood CO USA; ^4^ New Mexico Clinical Research & Osteoporosis Center and University of New Mexico Health Sciences Center Albuquerque NM USA; ^5^ INSERM UMR 1033, Université de Lyon, Hôpital Edouard Herriot Lyon France; ^6^ Hospital Universitario Quirón Salud Madrid, Universidad Europea Madrid Spain; ^7^ Centre for Metabolic Diseases, University of Sheffield Sheffield UK; ^8^ Mary Mackillop Institute for Health Research, Australian Catholic University Melbourne Australia

**Keywords:** DENOSUMAB, FRAX, HIP FRACTURE, OSTEOPOROSIS, OSTEOPOROTIC FRACTURE

## Abstract

The 3‐year placebo‐controlled FREEDOM (Fracture REduction Evaluation of Denosumab in Osteoporosis Every 6 Months) trial established the antifracture efficacy of denosumab in postmenopausal women with osteoporosis. The 7‐year open‐label extension demonstrated that denosumab treatment for up to 10 years was associated with low rates of adverse events and low fracture incidence. The extension lacked a long‐term control group, thus limiting the ability to fully evaluate long‐term efficacy. This analysis provides a quantitative estimate of the long‐term antifracture efficacy of denosumab based on two approaches: comparison with FRAX®‐ (Fracture Risk Assessment Tool‐) and virtual twin‐estimated 10‐year fracture rates. Subjects who were randomized to denosumab in the FREEDOM trial, continued into the Extension study, completed the 10‐year visit, and missed ≤1 dose in the FREEDOM trial and ≤1 dose in the Extension (*n* = 1278) were included in the analysis. The 10‐year observed cumulative incidence of major osteoporotic fracture (MOF) and hip fractures was compared with the 10‐year fracture probability predicted at baseline by FRAX, a computer‐based fracture risk algorithm, and with that estimated for a hypothetical cohort of 10‐year placebo controls (virtual twins). The observed 10‐year fracture incidence was lower than the 10‐year probability predicted by FRAX for both MOF (10.75% [95% CI, 9.05 to 12.46] versus 15.63% [95% CI, 15.08 to 16.18], respectively), and hip fractures (1.17% [95% CI, 0.58 to 1.76] versus 5.62% [95% CI, 5.28 to 5.97], respectively). The observed fracture incidence was also lower than the fracture rate estimated in a hypothetical cohort of 10‐year placebo controls for MOF (23.13% [95% CI, 17.76 to 28.87]; relative risk 0.49 [95% CI, 0.36 to 0.64]). These data support the long‐term efficacy of denosumab in reducing MOF and hip fractures in postmenopausal women with osteoporosis. © 2020 The Authors. *JBMR Plus* published by Wiley Periodicals, Inc. on behalf of American Society for Bone and Mineral Research.

## Introduction

Osteoporosis is a chronic, progressive condition that generally requires long‐term management. Denosumab is a fully human monoclonal antibody that binds to RANKL to inhibit osteoclast formation, function, and survival, and is approved for the treatment of postmenopausal women with osteoporosis at high risk for fracture.^1^ Compared with placebo, denosumab treatment significantly reduced vertebral, nonvertebral, and hip fracture risk; increased lumbar spine and total hip BMD; and reduced bone turnover markers in the pivotal, 3‐year FREEDOM (Fracture REduction Evaluation of Denosumab in Osteoporosis Every 6 Months) trial in postmenopausal women with osteoporosis.^2^ Continued denosumab administration over an additional 7 years of the FREEDOM Extension study was associated with low rates of adverse events, low fracture incidence compared with FREEDOM, and continued increases in BMD without plateau.^3^ However, the lack of a long‐term control group in the FREEDOM Extension study limits the ability to evaluate long‐term efficacy.

The purpose of this study was to provide a quantitative estimate of the long‐term antifracture efficacy of denosumab over 10 years in the absence of a long‐term control group. Thus, observed 10‐year cumulative fracture incidence during FREEDOM and its long‐term extension was compared with the estimated 10‐year probability of fracture using the FRAX® (fracture risk assessment tool) algorithm^4^ and the expected fracture rates in a theoretical group of long‐term placebo subjects using the virtual twin method. FRAX estimates the 10‐year probability of a major osteoporotic fracture (MOF: hip, clinical vertebral, forearm, or humerus) and hip fracture alone. The previously published^5^ virtual twin method uses a Poisson regression model to estimate fracture rates in a hypothetical cohort of placebo controls and has been used to estimate untreated fracture rates with alendronate^5^ and denosumab.^6^

## Methods

### Studies

The design of the FREEDOM (NCT00089791) trial and its Extension (NCT00523341) study have been published previously.^2^, ^3^ The FREEDOM trial was a phase 3, multinational, randomized, double‐blind, placebo‐controlled, 3‐year study that enrolled postmenopausal women 60 to 90 years old who had a lumbar spine or total hip *T*‐score < −2.5 at either location, but > −4.0 at both sites.

Subjects were randomized to receive either placebo or denosumab 60 mg s.c. once every 6 months (Q6M) for 3 years and were instructed to take at least 1 g of calcium and at least 400 IU of vitamin D supplements daily. All FREEDOM completers, those who completed their 3‐year visit, who did not discontinue investigational product, and did not miss more than one dose, were eligible to enter the 7‐year Extension study. In the Extension study, all participants were scheduled to receive denosumab 60 mg s.c. Q6M and were instructed to take daily calcium and vitamin D supplements.

Subjects who had received 10 years of denosumab treatment (3 years of the FREEDOM trial plus 7 years of the Extension study), completed the 10‐year visit, and missed no more than one dose in the FREEDOM trial and no more than one dose in the Extension study are included in these analyses (Supplementary Fig. S1). The FREEDOM trial and its Extension study were approved by the ethics committee or institutional review board at each site, and subjects provided written informed consent prior to any study‐related procedures.

### Assessment of fractures

An MOF was defined as any clinical vertebral, hip, forearm, or humerus fracture regardless of trauma severity. A clinical vertebral fracture is a new vertebral fracture assessed at either a scheduled or unscheduled visit and associated with any signs and/or symptoms indicative of a fracture, excluding any fracture associated with high trauma severity or a pathologic fracture. New vertebral fractures were identified as an increase of ≥1 Genant grade from the previous grade of 0, 1, or 2 noted in any vertebra from T4 to L4. Hip fractures were defined as those at the femoral neck, or the intertrochanteric or subtrochanteric region.

### FRAX‐predicted probability of fracture

Ten‐year MOF and hip fracture probabilities were assessed in all study completers who had received 10 years of denosumab treatment. Fracture probabilities used FREEDOM baseline characteristics and were generated with FRAX (version 3.11). FRAX included age, BMI, and femoral neck BMD. It also included yes/no data for the following risk factors: prior fracture (no adjustment for more than one fracture is provided in FRAX), parental hip fracture, daily alcohol consumption of ≥3 units, and current smoker status. Glucocorticoid use and rheumatoid arthritis were set to “no” as they were exclusion criteria for entry into the FREEDOM trial.

Missing data were simulated based on the conditional probability of the association of a risk factor with other risk factors (eg, age, BMI, or the dichotomous variables), using relationships between all the clinical risk factors including BMI and femoral neck BMD in the population‐based cohorts of more than 40,000 women used to develop FRAX.^7^

FRAX probabilities were computed with country‐specific data for the country where the subject was enrolled (Supplementary Table S1). Country models for Bulgaria, Latvia, and Serbia were unavailable; therefore, a surrogate was chosen that best reflected fracture probability based on the likelihood of similar incidences of fracture and mortality.

### Virtual twin approach

Because of the lack of a long‐term placebo group, fracture rates in a hypothetical cohort of 10‐year placebo controls (virtual twins) were estimated. Briefly, a virtual twin was generated for each subject with identical baseline characteristics, then fracture events during the FREEDOM trial and its Extension, assuming they remained on placebo, were predicted from a Poisson regression model built using placebo data from the FREEDOM trial. The Poisson regression model included baseline age, BMI, history of vertebral and nonvertebral fractures, *T*‐score, and smoking status (ever smokers versus nonsmokers) as predictors, and the logarithm of follow‐up time between randomization and end‐of‐trial of FREEDOM was used as offset.^8^ The expected fracture rates of virtual twins during the Extension study were estimated from the same Poisson model with updated values of the predictors at the Extension baseline. Predictors such as age, BMI, and smoking status were assumed not to be affected by denosumab treatment and their values for the virtual twins at Extension baseline were identical to those of the long‐term subjects. Fracture events during the FREEDOM trial and *T*‐score at the end of the trial were assumed to be affected by denosumab treatment and were modeled with FREEDOM data among crossover subjects using respective regression models: Incident fracture events during the FREEDOM trial for the virtual twins were predicted from the Poisson model described above; the expected mean total hip *T*‐score at the end of the trial was estimated by a linear regression model with FREEDOM baseline age, *T*‐score, and BMI as the predictors, and simulated as a normal random variable for the virtual twins. This process was repeated for 5000 bootstrap samples of both the long‐term and crossover subjects to obtain 95% CIs of estimated fracture rates.

### Analytic approach

Kaplan–Meier estimates of 10‐year observed cumulative incidence of MOF and hip fractures were determined.

## Results

Baseline characteristics were similar between the overall population of the denosumab arm of the FREEDOM trial (*N* = 3902) and the subset of subjects used for the present analysis, who completed 10 years of denosumab treatment (*N* = 1278; Table 1).

**Table 1:** Baseline Characteristics With Prevalence of Clinical Risk Factors

Characteristic	Received denosumab 60 mg Q6M	
	Subjects enrolled in FREEDOM trial *N* = 3902	10‐year completers^a^ *N* = 1278
Age (years), mean (SD)	72.3 (5.2)	70.8 (4.6)
BMI (kg/m^2^), mean (SD)	26.0 (4.1)	25.8 (4.0)
Femoral neck *T*‐score, mean (SD)	−2.2 (0.7)	−2.1 (0.7)
Prior fracture, *n* (%)	1985 (51)	655 (51)^b^
Parental hip fracture, *n* (%)	378 (10)	122 (10)
Alcohol ≥3 units daily, *n* (%)	67 (2)	28 (2)
Current smoker, *n* (%)	351 (9)	90 (7)
10‐year FRAX probability with BMD (%), mean (SD)		
MOF	16.1 (10.3)	15.6 (9.9)
Hip fracture	6.4 (7.2)	5.6 (6.2)

FRAX = fracture risk assessment tool; FREEDOM = (Fracture REduction Evaluation of Denosumab in Osteoporosis Every 6 Months); MOF = major osteoporotic fracture; Q6M = every 6 months.

a Subjects who had received 10 years of denosumab treatment (3 years of FREEDOM plus 7 years of the Extension study), completed the 10‐year visit, and missed no more than one dose in the FREEDOM trial and no more than one dose in the Extension study.

b At age ≥55 years.

**Figure 1:**
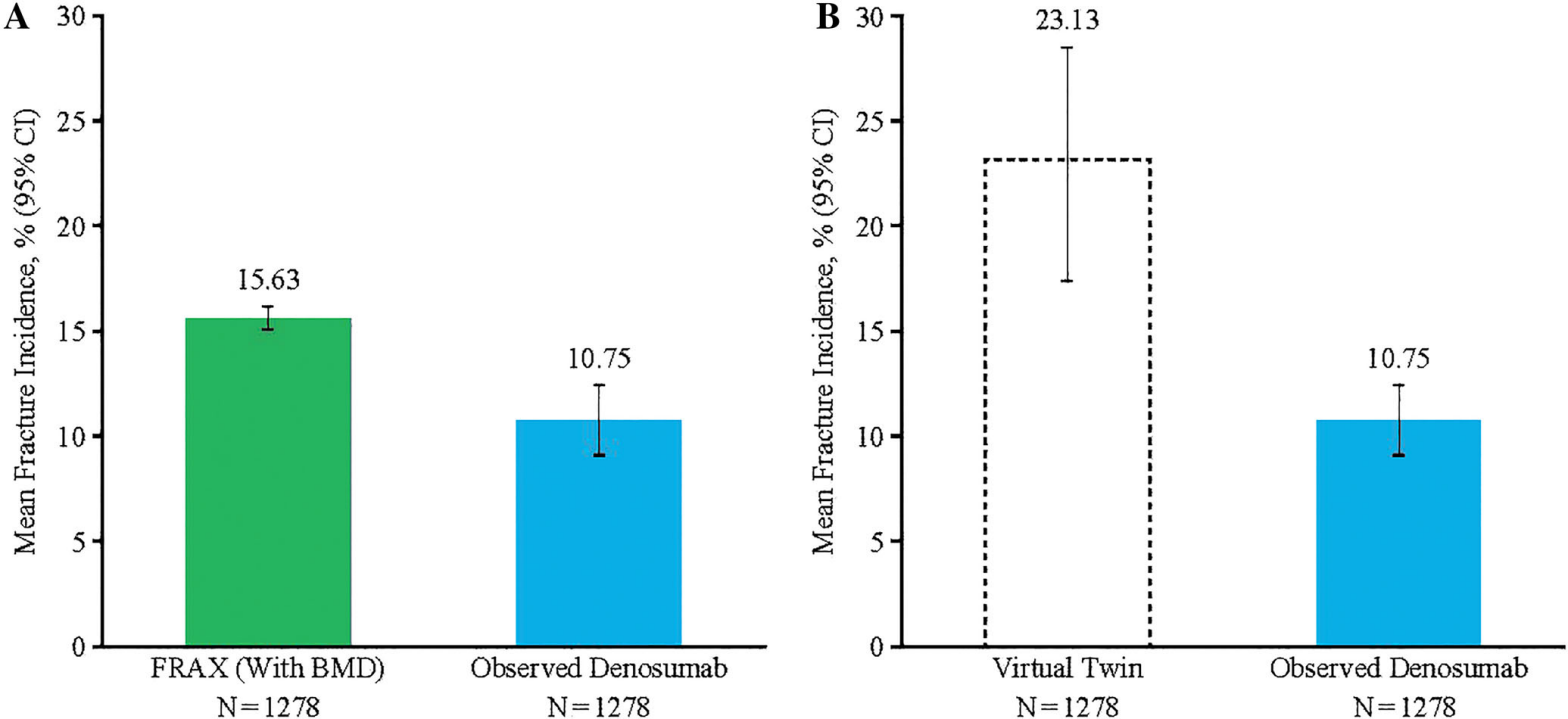
Major osteoporotic fracture incidence. Kaplan–Meier estimate of incidence at year 10, FRAX (fracture risk assessment tool) probability at the FREEDOM (Fracture REduction Evaluation of Denosumab in Osteoporosis Every 6 Months) trial baseline with BMD and virtual twin‐estimated incidence.

The 10‐year observed incidence of MOF (10.75%) was lower than both that predicted by FRAX (15.63%; Fig. 1 *A*) and that estimated for the virtual twin placebo group (23.13%; Fig. 1 *B*). Compared with the virtual twin placebo control group, denosumab treatment for 10 years was associated with a 51% MOF relative risk reduction (rate ratio: 0.49 [95% CI, 0.36 to 0.64]; Fig. 1 *B*).

**Figure 2:**
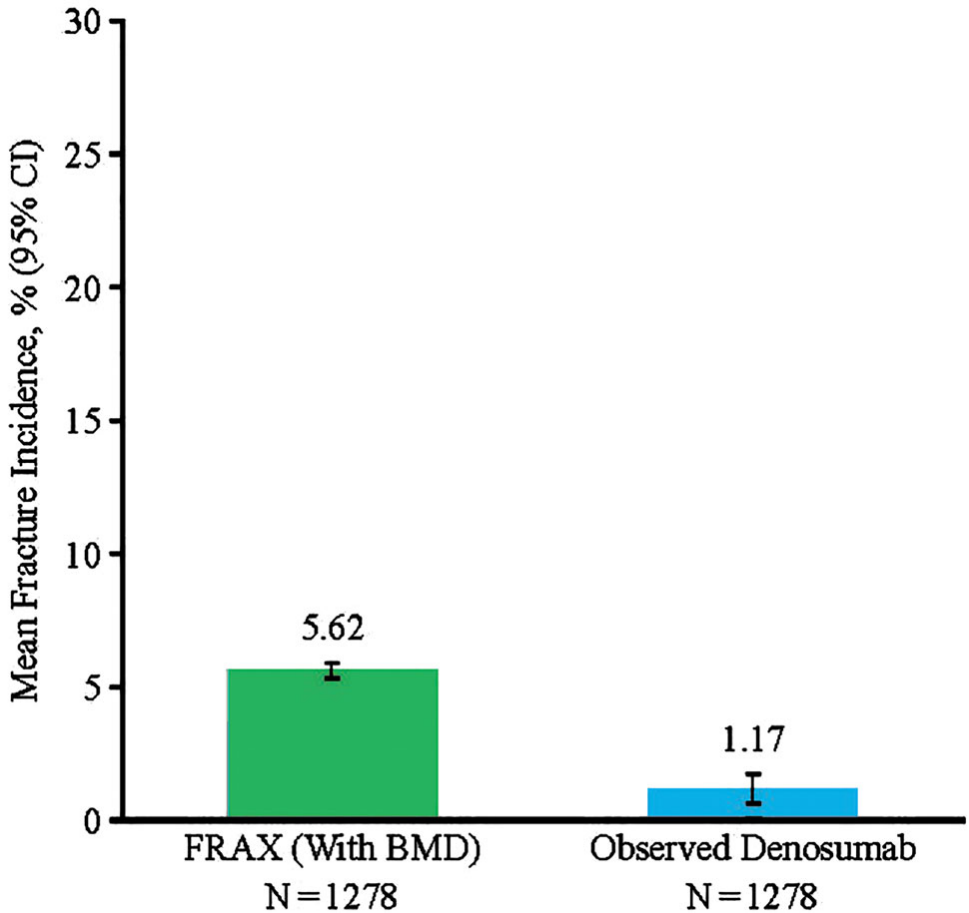
Hip fracture incidence. Kaplan–Meier estimate of incidence at year 10, FRAX (fracture risk assessment tool) probability at the FREEDOM (Fracture REduction Evaluation of Denosumab in Osteoporosis Every 6 Months) trial baseline with BMD and virtual twin‐estimated incidence. Virtual twin estimation of hip fractures was not calculated because of the low number of events.

Similarly, the 10‐year observed incidence of hip fracture (1.17%) was lower than that predicted by FRAX (5.62%; Fig. 2). Virtual twin estimation of hip fractures was not calculated because the low number of hip fracture events would lead to unreliable estimates based on the statistical models.

## Discussion

This analysis provides a quantitative estimate of the long‐term antifracture efficacy of denosumab in postmenopausal women with osteoporosis. Because all subjects received denosumab in the Extension study, two analytical approaches were used to compensate for the lack of a long‐term control group.

Denosumab treatment for 10 years was associated with lower incidences of MOF versus hypothetical placebo cohort using both FRAX and the virtual twin approach (11% for denosumab treatment, compared with 16% predicted using FRAX and 23% estimated for a virtual twin placebo group). This translates to a 51% lower observed MOF incidence with denosumab compared with that estimated for the virtual twin placebo cohort. A lower hip fracture incidence was also observed for denosumab than predicted using FRAX, although the low number of hip fracture events in FREEDOM prevented a reliable estimate using the virtual twin approach.

This analysis builds on previous data demonstrating that the low fracture incidence observed with denosumab in the initial 3‐year FREEDOM trial was maintained for up to 10 years.^3^ Strengths of this study include the use of two analytical approaches to further assess the efficacy of denosumab in the absence of actual long‐term placebo data. FRAX is a well‐accepted validated computer‐based algorithm used to estimate absolute fracture probabilities over 10 years^4^, ^9^, ^10^; the virtual twin approach is a statistical method used in previous analyses of denosumab and other osteoporosis medications.^6^ As previously stated, no adjustment for more than one fracture is provided in FRAX. Of *N* = 1278 subjects included in the 10‐year analysis, 518 (40.5%) had more than one prior fracture at age ≥ 55 years. Although we acknowledge that this could lead to an underestimated 10‐year fracture risk in these subjects, it is known that the greater the number of prior fractures, the greater the subsequent fracture risk. The virtual twin approach will factor in the impact of recurrent fractures in the FREEDOM 3‐year placebo group on subsequent fractures, but not over the extension period; therefore, this method will also have an intrinsic limitation.

Other limitations of this study include the lack of an actual placebo group over the long‐term duration of the FREEDOM extension period, and inclusion of only FREEDOM study completers, who had received denosumab for 10 years (approximately one‐third of the original denosumab cohort). Subjects discontinue participation in osteoporosis clinical trials for various reasons that may or may not be related to lack of efficacy^11^; therefore, the exclusion of subjects who discontinued treatment early can also be considered a strength of the study, as this allows efficacy to be estimated based on subjects who actually received denosumab for the full treatment period. Regardless, previous analyses found that the observed fracture incidence in the FREEDOM Extension study was not attributable to a healthy cohort of participants,^11^ and was reflective of the low fracture incidence data reported in the original trial (based on an intent‐to‐treat population).^2^, ^3^ Another limitation of the current study was the lack of county‐specific FRAX models for three countries, although the most appropriate surrogate model was chosen to best reflect fracture probability, this is a potential source of uncertainty. Finally, differences in estimated fracture rates between FRAX and virtual twin may be explained by methodological differences. FRAX employed country/region‐specific models using baseline prognostic factors. In contrast, the virtual twin method does not account for regional differences, but updates the prognostic factors at the Extension baseline to capture secular effects.

In conclusion, this analysis provides information on the long‐term efficacy of denosumab in postmenopausal women with osteoporosis utilizing both hypothetical placebo data and FRAX‐based probability data. Both analysis methods further support the concept that long‐term denosumab treatment for up to 10 years is associated with sustained fracture protection in postmenopausal women with osteoporosis.

## Disclosures

ES received royalties from UpToDate for peer review of denosumab. NP had employee and stock/stock options from Amgen Inc. at the time of the study. MM and SH have employee and stock/stock options from Amgen Inc. PDM has received grants/ research support from Alexion, Amgen Inc., Boehringer Ingelheim, Daiichi Sankyo, Eli Lilly, Immunodiagnostics, Merck, Merck Serrano, National Bone Health Alliance, Novartis, Radius, Roche Diagnostics, Regeneron, and Ultragenyx; he is a consultant for AgNovos, Amgen Inc., Eli Lilly, Merck, Radius Pharma, Roche, and Ultragenyx. EML has no direct income from potentially conflicting entities. His employer, New Mexico Clinical Research & Osteoporosis Center, has received research grants from Radius, Amgen, Mereo, and Bindex; income for service on scientific advisory boards or consulting for Amgen, Radius, Alexion, Sandoz, and Samsung Bioepis; service on speakersʼ bureaus for Radius and Alexion; project development for University of New Mexico; and royalties from UpToDate for sections on DXA, fracture risk assessment, and prevention of osteoporosis. He is a board member of the National Osteoporosis Foundation, International Society for Clinical Densitometry, and Osteoporosis Foundation of New Mexico. RC has received grants/research support from Amgen Inc., Merck, Pfizer, and Roche‐Chugai; he is a consultant for Amgen Inc., Pfizer, Ultragenyx, UCB, and BMS. He has received other financial or material support from AbbVie, Amgen Inc., Biogen, BMS, Eli Lilly, MSD, Pfizer, Roche‐Chugai, and Arrow. EJ‐G has received fees as a consultant, clinical investigator, and/or speaker from Amgen, AstraZeneca, Boehringer Ingelheim, FAES, Fresenius, GSK, Janssen, Lilly, MSD, Mundipharma, Novartis, Novo Nordisk, Pfizer, Sanofi, Shire, and UCB. JAK has received grants/research support from Amgen Inc., Eli Lilly, Medimaps, Radius Health, and Roche; he is a consultant for AgNovos, Eli Lilly, Medimaps, Radius Health, Theramex, and UCB.

## Supporting information


**Supplementary Table S1.** Countries included in the FRAX analysis with subjects enrolled in FREEDOM and who completed the 10‐year visit.

**Supplementary Figure S1.** Disposition of Subjects Included in this Analysis

## Data Availability

Qualified researchers may request data from Amgen clinical studies. Complete details are available at http://www.amgen.com/datasharing.
