# Supplementary material for: Estimation of Long‐Term Efficacy of Denosumab Treatment in Postmenopausal Women With Osteoporosis: A FRAX‐ and Virtual Twin‐Based Post Hoc Analysis From the FREEDOM and FREEDOM Extension Trials
Source: JBMR Plus. 2020 Feb 24;4(4):e10348. doi: 10.1002/jbm4.10348 (PMC7117843; doi:10.1002/jbm4.10348)
Supplement: Supplementary file 1 — Supplementary Table S1. Countries included in the FRAX analysis with subjects enrolled in FREEDOM and who completed the 10‐year visit. [file JBM4-4-e10348-s002.docx]

**Supplementary Material**

**Supplementary Table 1**. **Countries included in the FRAX analysis with subjects enrolled in FREEDOM and who completed the 10-year visit.**

| **Country** | **Race** | **Number of subjects**  **(N=1278)** |
| --- | --- | --- |
| Argentina | Hispanic or Latino | 25 |
| Argentina | White or Caucasian | 20 |
| Australia | White or Caucasian | 10 |
| Austria | White or Caucasian | 22 |
| Belgium | Other | 1 |
| Belgium | White or Caucasian | 21 |
| Brazil | Black or African American | 9 |
| Brazil | Hispanic or Latino | 22 |
| Brazil | White or Caucasian | 48 |
| Bulgaria | White or Caucasian | 6 |
| Canada | Japanese | 1 |
| Canada | White or Caucasian | 20 |
| Czech Republic | White or Caucasian | 102 |
| Denmark | White or Caucasian | 310 |
| Estonia | White or Caucasian | 101 |
| Finland | White or Caucasian | 8 |
| France | White or Caucasian | 19 |
| Germany | White or Caucasian | 31 |
| Greece | White or Caucasian | 4 |
| Hungary | White or Caucasian | 33 |
| Italy | White or Caucasian | 31 |
| Latvia | White or Caucasian | 17 |
| Lithuania | White or Caucasian | 16 |
| Mexico | Hispanic or Latino | 22 |
| New Zealand | White or Caucasian | 8 |
| Norway | White or Caucasian | 32 |
| Poland | White or Caucasian | 128 |
| Serbia | White or Caucasian | 2 |
| Slovakia | White or Caucasian | 12 |
| Spain | Hispanic or Latino | 1 |
| Spain | White or Caucasian | 45 |
| Sweden | White or Caucasian | 24 |
| Switzerland | White or Caucasian | 11 |
| United Kingdom | White or Caucasian | 87 |
| United States | Hispanic or Latino | 5 |
| United States | White or Caucasian | 24 |

**Supplementary Figure 1 Legend. Disposition of Subjects Included in this Analysis**
