# Supplementary figures and images for: Estimation of Long‐Term Efficacy of Denosumab Treatment in Postmenopausal Women With Osteoporosis: A FRAX‐ and Virtual Twin‐Based Post Hoc Analysis From the FREEDOM and FREEDOM Extension Trials
Source: JBMR Plus. 2020 Feb 24;4(4):e10348. doi: 10.1002/jbm4.10348 (PMC7117843; doi:10.1002/jbm4.10348)

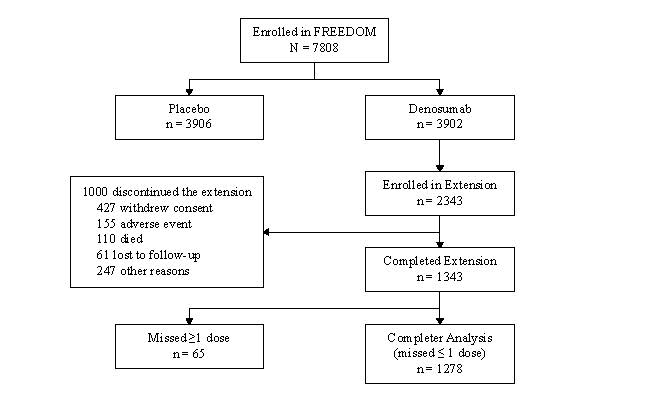

Supplement: Supplementary file 2 — Supplementary Figure S1. Disposition of Subjects Included in this Analysis [file JBM4-4-e10348-s001.jpg]
